# Study on Thermal Stability, Phase Transition Characteristics, and Pyrolysis Product Distributions of Long-Chain n-Alkanes (C12–C15)

**DOI:** 10.3390/molecules31132291

**Published:** 2026-07-01

**Authors:** Zengbo Ke, Yang Zhan, Mei Bai, Fengying Chen, Chengfang Qiao

**Affiliations:** 1 College of Chemical Engineering and Modern Materials, Shaanxi Key Laboratory of Comprehensive Utilization of Tailings Resources, Shangluo University, Shangluo 726000, China; kezengbo2021@163.com (Z.K.); baimei0508@163.com (M.B.); 2 College of Chemistry and Chemical Engineering, Shaanxi University of Science and Technology, Xi’an 710016, China

**Keywords:** long-chain n-alkanes, multiscale theoretical simulation, pyrolysis mechanism, phase transition

## Abstract

This study employs a multiscale theoretical approach to systematically investigate the thermal stability, phase transition characteristics, and pyrolysis product distributions of four long-chain n-alkanes ranging from n-dodecane to n-pentadecane (C12–C15). At the electronic structure level, density functional theory calculations reveal that with increasing chain length, the HOMO–LUMO gap narrows monotonically from 8.87 eV to 8.77 eV and global softness increases, indicating enhanced electronic responsiveness to thermal perturbation. Molecular electrostatic potential analysis shows decreasing surface potential variance and 100% nonpolar surface area across all species, confirming that intermolecular interactions are exclusively governed by London dispersion forces. At the condensed-phase level, semiempirical quantum-based molecular dynamics (xTB-MD) simulations at 3500 K over 6 ps trajectories reveal qualitative chain-length-dependent initial bond-breaking patterns: C2 species appear prominently among early fragments for C12–C15 systems, with medium-sized fragments (C3, C4) becoming increasingly prevalent and C1 species relatively less prominent as chain length grows. This work provides an integrated “electronic structure-condensed phase transition-pyrolysis kinetics” perspective, offering precise theoretical insights and critical benchmark data for the pyrolysis mechanisms of long-chain n-alkanes.

## 1. Introduction

With the continuous growth of global energy demand and increasingly stringent environmental regulations, the development of efficient and clean alternative fuels for aviation kerosene and diesel has become a central focus in energy chemistry [1,2]. As the primary constituents of both aviation kerosene and diesel, n-alkanes dictate combustion efficiency and the formation mechanisms of pollutants such as soot through their intrinsic combustion and pyrolysis characteristics [3,4]. Long-chain n-alkanes ranging from C12 to C15 are particularly significant in this context: they not only serve as representative surrogates for real fuels but also constitute essential model compounds for the construction of detailed chemical kinetic mechanisms [5,6]. A thorough understanding of the microscopic decomposition behavior, the site-selectivity of initial bond cleavage, and the subsequent evolution pathways of product distributions under high-temperature extreme conditions is therefore of critical importance for optimizing fuel formulations and suppressing the formation of soot precursors.

Although experimental techniques such as single-photon vacuum ultraviolet (VUV) have achieved significant progress in acquiring macroscopic reaction rates and major product distributions, real-time capture of extremely short-lived radical intermediates at the atomic scale and tracking the dynamic processes of chemical bond breaking under high-temperature conditions remain formidable challenges [7]. Experimental approaches often struggle to resolve isomeric products with similar mass-to-charge ratios and are inherently incapable of directly observing reaction transition states or transient electronic structure rearrangements [8]. Consequently, theoretical and computational methods rooted in quantum mechanics and statistical mechanics have emerged as indispensable tools for elucidating these microscopic mechanisms [9,10]. In recent years, molecular dynamics simulations have been extensively applied to fuel pyrolysis research owing to their unique capability of visualizing atomic trajectories [11,12,13]. For instance, Liu et al. [14] employed reactive force field (ReaxFF) molecular dynamics to successfully unravel the four-stage process of soot nanoparticle formation from n-decane pyrolysis, clarifying growth mechanisms such as Hydrogen-Abstraction-Carbon-Addition (HACA). Similarly, Wang et al. [15] have utilized this approach to investigate the pyrolysis behavior of phenolic resins and Polyoxymethylene Dimethyl Ethers (PODE_n_), respectively, yielding valuable insights into the evolution of small-molecule products. However, although traditional reactive force field methods offer computational efficiency advantages for handling large-scale systems, their potential parameters are typically fitted against limited training sets. A precision bottleneck thus persists when treating phenomena involving complex electronic reorganization, excited-state effects, or accurate bond dissociation barriers under specific chemical environments, potentially leading to the misidentification of critical reaction pathways [16,17].

To overcome these limitations and obtain dynamic reaction information with enhanced fidelity, this study introduces a state-of-the-art semi-empirical method—GFN1-xTB [18]—developed within the framework of density functional tight binding (DFTB). This approach maintains an accuracy approaching that of first-principles calculations while substantially reducing computational cost, thereby enabling long-time-scale first-principles molecular dynamics simulations on systems comprising tens of atoms. In contrast to conventional empirical force fields, GFN1-xTB explicitly accounts for variations in the electronic structure, allowing for more reliable predictions of bond-breaking sequences and radical formation processes [19].

Herein, we present a systematic multiscale theoretical investigation targeting four long-chain n-alkanes from n-dodecane (C12) to n-pentadecane (C15). Rigorous structural optimizations and single-point energy calculations were initially performed for the four molecules using density functional theory (DFT). This not only yielded well-defined initial geometric configurations but also enabled in-depth analyses of frontier molecular orbital (FMO) distributions and molecular surface electrostatic potential (ESP) characteristics, thereby establishing a robust electronic-structure foundation for the subsequent dynamics simulations. To validate the modeling approach and probe macroscopic thermophysical properties, large-scale simulation boxes of 50 × 50 × 50 Å^3^ containing 50 molecules were constructed. Classical molecular dynamics simulations were carried out using the GROMACS (2018) package in conjunction with RESP2_0.5_ charges. By examining the equilibrated phase at 298 K and analyzing the temporal evolution of temperature and density during linear heating from 0 K to 2500 K, the phase transition behaviors of the four compounds were preliminarily characterized. Building upon the DFT calculations, medium-scale boxes of 30 × 30 × 30 Å^3^ containing 10 molecules were prepared using Packmol and GROMACS for subsequent CP2K (2025.1) simulations. Semi-empirical molecular dynamics simulations employing the high-accuracy GFN1-xTB method were conducted under the control of a CSVR thermostat for a total of 30,000 steps. Through these high-fidelity dynamic simulations, we elucidate the differential bond-cleavage patterns among the four long-chain alkanes during pyrolysis and systematically compare the influence of chain length on the distribution of pyrolysis products. From a panoramic perspective spanning electronic structure to atomistic dynamics, this work aims to provide a more precise theoretical interpretation of the pyrolysis mechanisms of long-chain n-alkanes and to deliver critical benchmark data for the future development of high-fidelity detailed chemical kinetic mechanisms.

## 2. Results and Discussion

### 2.1. FMOs and ESP

**Figure 1:**
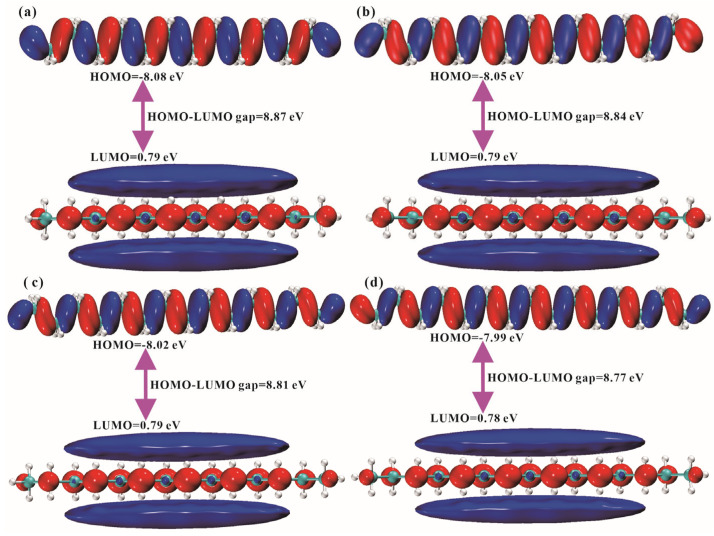
Energy-level diagrams of the frontier molecular orbitals (HOMO and LUMO) for molecules C12–C15: (**a**) C12, (**b**) C13, (**c**) C14, (**d**) C15. The blue and red isosurfaces represent the negative and positive phases of the orbital wavefunctions, respectively.

Parameters derived from frontier molecular orbital (FMO) analysis based on density functional theory calculations systematically reveal the evolution of electronic structure and the underlying regulatory mechanisms governing thermal stability and reactivity for C12–C15 long-chain n-alkanes. Frontier orbital analysis offers insights into the molecular reactivity descriptors of these compounds, enhancing our understanding of their chemical properties [20,21,22]. Key descriptors such as electronegativity (*χ*) and chemical hardness (*η*) can be calculated using the formulas *χ* = (*I* + *A*)/2, and *η* = (*I* − *A*)/2, where *I* (ionization energy) and *A* (electron affinity) are related to the HOMO and LUMO energies (*I* = −*E*_HOMO_, *A* = −*E*_LUMO_). The chemical potential (*μ*) is the negative of the electronegativity (*μ* = −*χ*), while the chemical softness (*σ*) and electrophilicity index (*ω*) are given by *σ* = 1/(2*η*) and *ω* = *χ*^2^/(2*η*). As summarized in Table 1, with increasing chain length from C12 to C15, the highest occupied molecular orbital energy (*E*_HOMO_) rises monotonically from −8.08 eV to −7.99 eV, while the lowest unoccupied molecular orbital energy (*E*_LUMO_) decreases slowly from 0.79 eV to 0.78 eV, resulting in a strictly monotonic narrowing of the HOMO-LUMO gap (8.87 → 8.77 eV). Although the absolute magnitude of this gap contraction is modest—corresponding to an average decrease of approximately 0.035 eV per -CH_2_- unit—the monotonic trend carries clear physical significance: it indicates that the kinetic stability of the molecules against external thermal perturbation and electronic excitation weakens systematically with increasing chain length. Derived chemical reactivity descriptors further corroborate this trend. The decrease in ionization energy (*I*) (8.08 → 7.99 eV) reflects an enhanced propensity for electron loss; the global hardness (*η*) decreases from 4.44 eV to 4.39 eV, collectively pointing to a gradual enhancement of the molecular polarizability response. The electrophilicity index (ω) remains nearly constant across all investigated systems (C12–C15), ranging from 1.48 to 1.50 eV, indicating that the change in chain length has no substantial effect on the overall electrophilicity of the molecules. The initial reactivity of n-dodecane is essentially consistent with that of longer-chain alkanes under electrophilic or radical attack conditions. As illustrated by the orbital phase distributions in Figure 1, the HOMO and LUMO electron densities are predominantly delocalized over the σ-bonding and σ*-antibonding orbitals of the carbon skeleton, with a relative decrease in terminal-group contributions as the chain length increases. These electronic structural features provide a microscopic theoretical foundation for understanding the chain-length dependence of C-C bond homolysis site selectivity during high-temperature pyrolysis: a narrower gap and higher softness imply a reduced electronic reorganization barrier, which may in turn influence the relative abundance of short-chain fragments in the pyrolysis product distribution.

**Figure 2:**
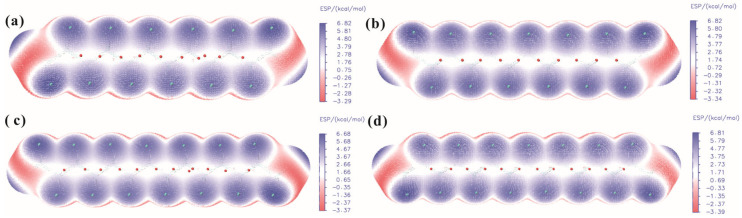
Electrostatic potential maps on molecular surfaces: (**a**) C12; (**b**) C13; (**c**) C14; (**d**) C15.

**Figure 3:**
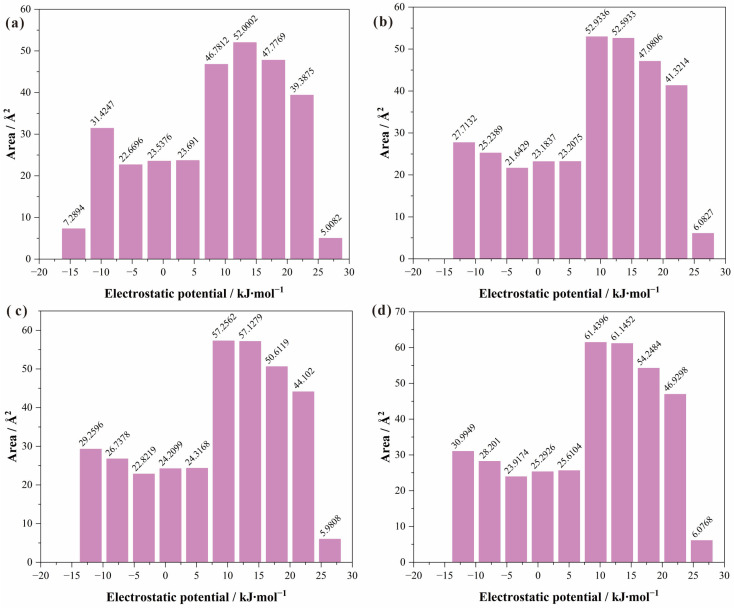
Electrostatic potential (ESP) distributions of surface areas as a function of energy for the four molecules: (**a**) C12; (**b**) C13; (**c**) C14; (**d**) C15.

Molecular surface electrostatic potential (ESP) analysis elucidates the electronic origins of condensed-phase structure and phase transition behavior in long-chain n-alkanes from the perspective of non-covalent interactions. As presented in Table 2, the ESP minimum (most negative potential) shifts monotonically from −13.81 kJ·mol^−1^ to −14.19 kJ·mol^−1^, while the ESP maximum (most positive potential) remains essentially stable within a narrow range of 28.48–28.54 kJ·mol^−1^. The overall average ESP value decreases slightly from 8.41 kJ·mol^−1^ to 8.37 kJ·mol^−1^ with increasing chain length. The overall variance declines from 57.66 (kJ·mol^−1^)^2^ to 56.18 (kJ·mol^−1^)^2^, unequivocally demonstrating that the degree of inhomogeneity in the molecular surface potential distribution continuously diminishes as the chain lengthens—that is, the potential distribution becomes increasingly flat and isotropic. Systematic variations in the charge balance parameter and the internal charge separation parameter further reinforce this conclusion. Most critically, the nonpolar surface area fraction (|ESP| ≤ 41.84 kJ·mol^−1^) is 100% for all four compounds, whereas the polar surface area fraction is 0%, conclusively establishing that the nature of intermolecular interactions among C12–C15 molecules is governed exclusively by London dispersion forces [23,24], with electrostatic contributions to molecular recognition and crystal packing being entirely negligible. The ESP color mapping in Figure 2 visually demonstrates that the potential across the main body of the carbon chain is nearly neutral, with only a faint positive potential (blue) at the terminal hydrogen atoms and negative potential (red) confined to the C-C bond axis regions. This highly flat and isotropic ESP distribution pattern carries profound implications for condensed-phase behavior: within a crystalline environment, the local electrostatic environment of an alkane molecule undergoes negligible fluctuations during long-axis rotation, terminal-group torsion, or lateral displacement, resulting in exceptionally low intermolecular energy barriers to conformational changes. This electronic structural characteristic provides direct microscopic evidence for the rich solid–solid rotational phases and premelting behavior exhibited by long-chain n-alkanes upon heating: precisely because of the extreme flatness of the surface potential, the additional dispersion energy contributed by chain elongation cooperatively locks molecular translational degrees of freedom while simultaneously preserving dynamic flexibility for long-axis rotation and local conformational torsion. Figure 3 illustrates the molecular surface electrostatic potential (ESP) area distributions for four molecules (a–d, corresponding to carbon chain lengths C12–C15), with ESP values (kJ·mol^−1^) on the abscissa and area (Å^2^) on the ordinate. All ESP values fall within the range of −16.74 to 29.29 kJ·mol^−1^, which is far below the nonpolar threshold of 41.84 kJ·mol^−1^, indicating that the molecular surfaces are entirely composed of nonpolar regions with no polar contribution, consistent with the conclusions of Table 2.

### 2.2. Phase Transition Simulations

**Figure 4:**
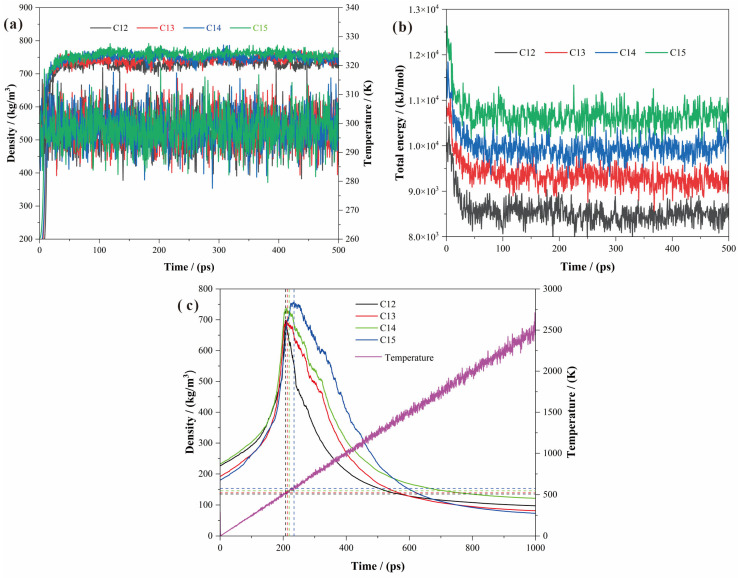
Validation of equilibrium and heating protocols for the molecular dynamics simulations of C12–C15 long-chain n-alkanes: (**a**) Temperature and density as a function of simulation time during the NVT production phase; (**b**) Total energy as a function of simulation time during the NVT production phase; (**c**) Density and temperature as a function of simulation time during programmed heating. The vertical dashed lines correspond to the peak positions of each curve, and the horizontal dashed lines corre-spond to the corresponding temperatures.

As illustrated in Figure 4a,b, C12–C15 n-alkanes exhibit robust thermodynamic equilibration during the production phase of molecular dynamics simulations. The average temperature of all four systems is precisely maintained near 298 K (error: 0.11–0.18 K), while the temperature root-mean-square deviation (RMSD) decreases monotonically with increasing chain length (6.31 K → 5.81 K). This trend parallels the reduced surface electrostatic potential variance discussed in Section 2.1. While a direct causal link cannot be established from equilibrium MD alone, it is plausible that the more uniform charge distributions in longer chains—arising from σ-conjugative delocalization across extended C-C backbones, charge dilution over a larger molecular surface, and enhanced conformational averaging that suppresses local dipole heterogeneity—may facilitate more efficient dissipation of thermal fluctuations. Concurrently, the average density increases monotonically with carbon number (717.7 → 748.5 kg·m^−3^), accompanied by a marked reduction in density fluctuation RMSD [25] (85.8 → 62.3 kg·m^−3^). This observation is physically reasonable and suggests that the cumulative strengthening of London dispersion forces likely enhances condensed-phase packing rigidity. Furthermore, the total drift [26] values for all systems are uniformly negative with negligible absolute magnitudes (−0.25 to −1.28 K), indicating no significant systematic deviation in energy or temperature over the simulation duration, thereby further corroborating the complete release of initial configurational stress and the high reliability of the thermostat coupling algorithm. The absolute average total energy increases monotonically with chain length (C12: 8548.12 kJ·mol^−1^; C13: 9363.22 kJ·mol^−1^; C14: 9978.11 kJ·mol^−1^; C15: 10684.90 kJ·mol^−1^), reflecting the cumulative increase in molar internal energy arising from greater atomic count and intramolecular degrees of freedom. Meanwhile, the total energy remains stable throughout the production run with highly consistent RMSD values across all systems (286–301 kJ·mol^−1^), demonstrating thorough equilibration and establishing a reliable steady-state baseline for subsequent programmed heating simulations. Figure S1 illustrates the uniform molecular packing of all four compounds (C12–C15) within the simulation cells, validating the reasonableness of the prepared models.

As shown in Figure 4c, the density profiles during programmed heating display a clear nonlinear response: following an initial gradual increase, each system undergoes an abrupt step-like drop at a characteristic critical temperature, signifying a dramatic transition from the condensed phase to a low-density phase. The characteristic transition temperatures (T_trans_), determined from the maximum of the density curve prior to the abrupt drop, are 522 K (C12), 534 K (C13), 556 K (C14), and 588 K (C15). Although these simulated values exceed experimental boiling points (e.g., 489 K for n-dodecane) by approximately 30–45 K—attributable to the absence of a free surface and additional evaporation energy barriers under periodic boundary conditions—the monotonic increase of T_trans_ with chain length (average increment of ~22 K per –CH_2_– unit) aligns excellently with experimental trends. A detailed comparison of simulated and experimental transition temperatures is provided in Table S1 (Supplementary Materials). This agreement supports the reliability of the force field parameters in capturing the cumulative effect of intermolecular dispersion interactions. From a physical perspective, it is reasonable to interpret that longer carbon chains provide greater van der Waals contact area and more dispersion interaction sites, significantly elevating the cohesive energy density, which plausibly necessitates greater thermal driving force to escape from the intermolecular attractive potential well for phase transition.

The statistical parameters reported in Table 3, Table 4 and Table 5 were derived from the production phase trajectories. Specifically, the “Error Estimate” represents the standard error of the mean (SEM), calculated by block averaging the trajectory data to assess the convergence of the ensemble average. The “RMSD” (root-mean-square deviation) quantifies the magnitude of thermal fluctuations around the mean value, computed as the square root of the variance of instantaneous values relative to the time-averaged mean. These metrics collectively characterize the stability and sampling quality of the equilibrated systems.

### 2.3. Initial Bond Cleavage and Early-Stage Dynamics

**Figure 5:**
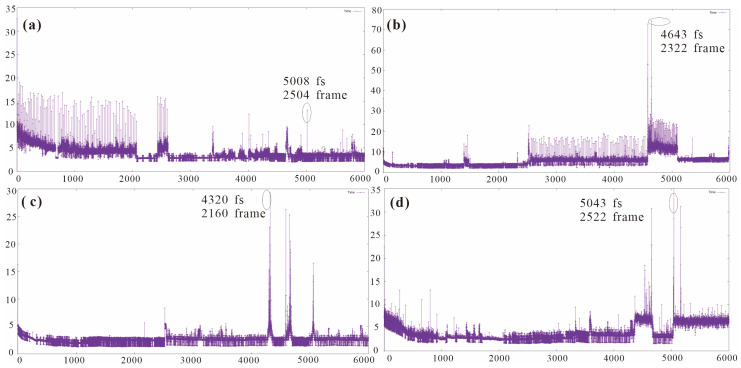
Transient fragment counts during the initial decomposition phase and instantaneous computational wall time per molecular dynamics step in xTB molecular dynamics simulations of C12–C15 long-chain n-alkanes at 3500 K. The instantaneous wall time per step is shown; sharp peaks in the time profile correspond to bond breaking or formation events that trigger electronic density reorganization and directly lead to changes in the transient counts of various carbon-containing fragments. Panels (**a**), (**b**), (**c**), and (**d**) correspond to C12, C13, C14, and C15, respectively.

**Figure 6:**
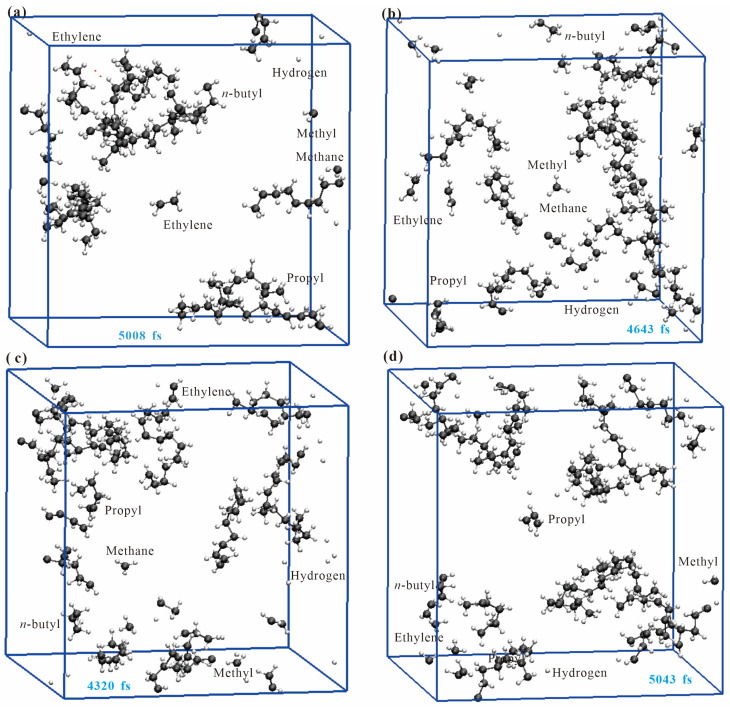
Representative snapshots of transient fragment counts during the initial decomposition phase of C12–C15 long-chain n-alkanes in xTB dynamics simulations. (**a**) C12 at frame 2504; (**b**) C13 at frame 2322; (**c**) C14 at frame 2160; (**d**) C15 at frame 2522. The notation Cn (*n* ≥ 1) denotes fragments containing n carbon atoms, and C0 represents molecular hydrogen (H_2_).

The xTB molecular dynamics (xTB-MD) simulations presented herein employ a single 6 ps trajectory per system at 3500 K. Given the highly stochastic nature of bond cleavage at this temperature and the limited sampling duration, the reported fragment counts represent transient, non-equilibrated observations of initial bond-breaking events rather than statistically converged product distributions. No independent replicate trajectories or uncertainty estimates are available. Results should therefore be interpreted exclusively as qualitative insights into early-stage reactivity trends and site-selectivity patterns, not as quantitative pyrolysis yields. To elucidate the microscopic mechanisms of chemical bond cleavage during the initial stages of pyrolysis in C12–C15 long-chain n-alkanes, xTB-MD simulations were performed at 3500 K using the CP2K program package. Figure 5 presents the instantaneous computational wall time per molecular dynamics step throughout the simulation. The wall-time profile exhibits multiple sharp pulse-like spikes, which correlate directly with abrupt increases in the number of self-consistent field (SCF) iterations required for convergence. When chemical bonds break or form, the electron density undergoes substantial reorganization, necessitating additional SCF iterations to reach convergence; thus, these wall-time spikes serve as sensitive probes for reactive events. The dense and aperiodic distribution of wall-time spikes across all four systems indicates that C-C and C-H bond cleavage at 3500 K proceeds in a highly stochastic manner. Figure 6 displays representative snapshots of pyrolysis fragments for the four compounds at specific simulation frames: C12 at frame 2504, C13 at frame 2322, C14 at frame 2160, and C15 at frame 2522. Extensive bond scission is observed in all systems, yielding a variety of primary products including ethylene, methyl radical, methane, n-propyl radical, n-butyl radical, and other carbon-containing fragments of varying carbon number (denoted as Cn, where n represents the number of carbon atoms in the species), as well as molecular hydrogen (designated as C0). Notably, C2 species constitute a significant fraction of the products in all systems, consistent with the classical Rice–Kossiakoff mechanism wherein *β*-scission preferentially generates ethylene [27]. The relatively higher proportions of C3 and C4 fragments in the C14 and C15 systems suggest that longer carbon chains exhibit a greater tendency to form medium-length alkyl radicals during the primary cracking stage. This chain-length dependence aligns with the electronic structure evolution revealed by the narrowing HOMO-LUMO gap and increasing global softness described in Section 2.1: the enhanced polarizability response of longer chains leads to a more delocalized distribution of C-C bond cleavage sites, thereby generating a richer spectrum of primary fragments.

**Figure 7:**
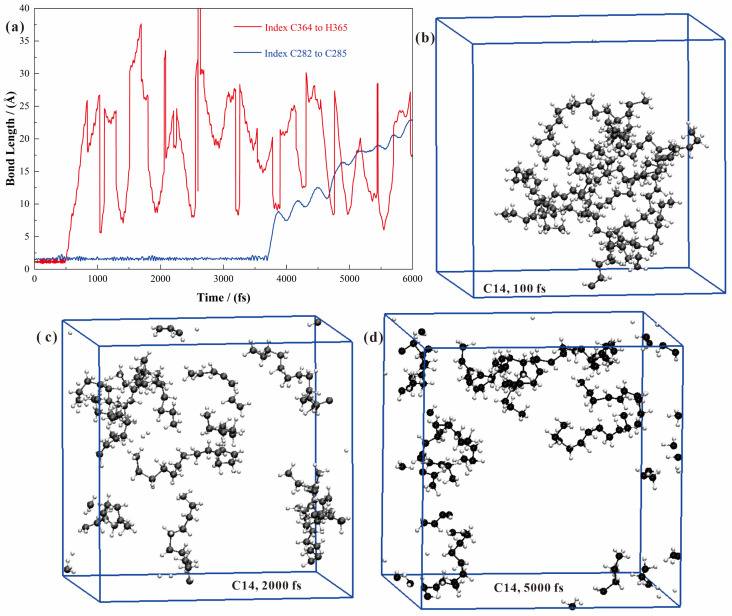
Geometric evidence and visual evolution of bond cleavage in the C14 system during xTB-MD simulations at 3500 K. (**a**) Time-evolution profiles of specific bond lengths: the blue curve represents the C–C bond between atoms 282 and 285, while the red curve represents the C–H bond between atoms 364 and 365. The abrupt elongation of these bonds beyond their dissociation thresholds (indicated by dashed lines) provides direct geometric confirmation of chemical reactions, replacing the previously used computational wall-time metric. (**b**–**d**) Representative snapshot sequences illustrating the fragmentation process of the C14 system at distinct time intervals: (**b**) 100 fs, showing the initial intact state with 429 bonds and 11 fragments; (**c**) 2000 fs, displaying intermediate decomposition with 401 bonds and 39 fragments; and (**d**) 5000 fs, revealing extensive pyrolysis with 386 remaining bonds and 55 distinct fragments. The reacting molecules are highlighted in ball-and-stick representation, while surrounding species are rendered as transparent lines to emphasize the reaction sites.

To rigorously validate the occurrence of chemical reactions and address the limitations of using computational wall time as a sole indicator, we performed a comprehensive geometric analysis of bond length evolution. As illustrated in Figure 7a, we tracked the interatomic distances of representative C–C and C–H bonds in the C14 system. The blue curve shows the C–C bond length between atoms 282 and 285, which remains stable below 2.1 Å until 3704 fs. Subsequently, it undergoes an abrupt elongation exceeding 3 Å after 3730 fs, unequivocally signaling C–C bond scission. Similarly, the red curve depicts the C–H bond between atoms 364 and 365, which maintains stability below 1.7 Å for the first 500 fs before stretching beyond 2 Å after 510 fs, eventually reaching distances greater than 42 Å, confirming C–H bond cleavage. Complementing this quantitative data, Figure 7b–d present visual snapshots of the C14 system at 100 fs, 2000 fs, and 5000 fs, respectively. Statistical analysis of these snapshots reveals a progressive decrease in total bond count (from 429 to 386) and a corresponding increase in fragment number (from 11 to 55), visually corroborating the stochastic nature of bond breaking and the subsequent formation of diverse pyrolysis products. This combined geometric and visual evidence establishes a robust foundation for identifying reaction events in our xTB-MD trajectories.

**Figure 8:**
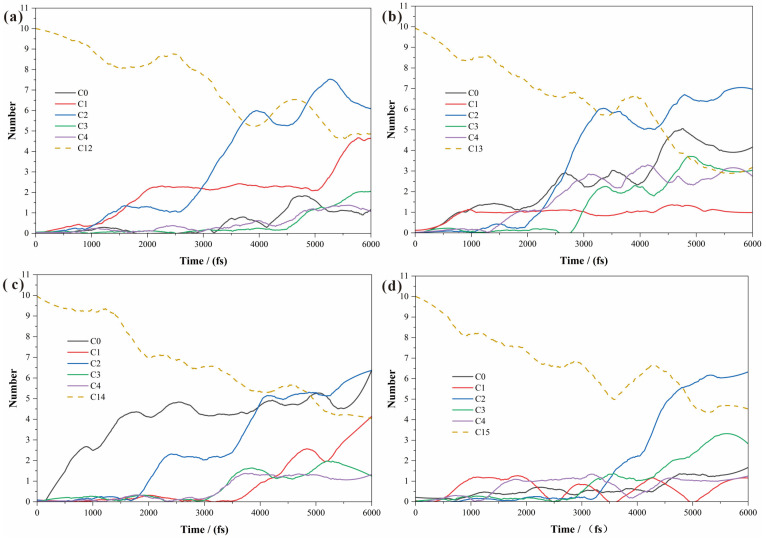
Temporal evolution of transient fragment counts of various carbon-containing fragments during the initial decomposition phase of C12–C15 long-chain n-alkanes in xTB dynamics simulations at 3500 K. The figure shows the instantaneous appearance and decay of various carbon-containing species (C_1_, C_2_, C_3_ and C_4_, as well as H_2_) during the initial high-temperature pyrolysis process, reflecting non-equilibrium early-stage stochastic bond-breaking events rather than time-averaged equilibrium product distributions. Panels (**a**), (**b**), (**c**), and (**d**) correspond to C12, C13, C14, and C15, respectively.

Figure 8 quantitatively summarizes the temporal evolution of the populations of various carbon-containing fragments over the time window of 5000–6000 fs. Based on the time-averaged values of each species population curve, the abundance rankings of pyrolysis products for the four alkanes are as follows: for C12, C1 > C2 > C4 > C3 > C0; for C13, C2 > C0 > C3 > C4 > C1; for C14, C2 > C0 > C1 > C3 > C4; and for C15, C2 > C3 > C0 > C4 > C1. A systematic analysis of these results reveals clear chain-length-dependent trends. C2 species rank first in abundance for the three longer-chain systems (C13–C15) and are only marginally surpassed by C1 in the case of C12, unequivocally demonstrating that *β*-scission to ethylene constitutes the dominant pyrolysis pathway and that the relative contribution of this pathway increases with chain length. The abundance of molecular hydrogen (C0) rises monotonically with chain length, a consequence of the greater number of secondary hydrogen atoms—which possess lower C-H bond dissociation energies—in longer chains. The relative abundance of C1 species exhibits a declining trend with increasing chain length: it ranks first for C12, drops to third for C14, and falls outside the top two for both C13 and C15. This trend arises because terminal bond cleavage carries a higher statistical weight in shorter chains and secondary degradation pathways are shorter, whereas the longer fragments generated by primary scission in longer chains do not undergo complete degradation within the accessible simulation timescale, leading to a decreased relative yield of C1 species and correspondingly increased proportions of medium-sized fragments such as C2, C3, and C4. The elevation of C3 species to the second most abundant fragment in the C15 system further corroborates this interpretation. Collectively, the xTB-MD simulations validate the classical free-radical pyrolysis mechanism from the microscopic perspective of electronic structure and chemical bond cleavage, quantitatively reveal the migration trends in the pyrolysis product spectrum with increasing chain length, and provide critical reaction kinetic evidence that underpins the multiscale theoretical framework encompassing thermal stability, phase transition characteristics, and pyrolysis product distributions.

## 3. Computational Details

### 3.1. Quantum Chemical Calculations

Initial molecular structures of the four n-alkanes (n-dodecane, n-tridecane, n-tetradecane, and n-pentadecane) were optimized using density functional theory (DFT) [28]. All calculations were performed with the Gaussian 16 [29] and GaussView 6.0 [30]. Geometry optimizations were carried out at the B3LYP/def2TZVP level of theory [31,32] with Grimme’s D3 dispersion correction [33]. Single-point energy calculations were conducted at the same theoretical level to obtain total molecular energies. Based on the optimized wavefunctions, frontier molecular orbital (FMO) [34,35] energies and spatial distributions were analyzed, and molecular surface electrostatic potential (ESP) [36] parameters—including global minima, global maxima, average potential, and molecular polarity indices—were computed using the Multiwfn 3.8 program package [37,38]. All visualizations were generated with VMD 1.9.3 [39].

### 3.2. Classical Molecular Dynamics Simulations

Classical molecular dynamics (MD) simulations [40,41] were performed using GROMACS 2018 [42]. Initial configurations containing 50 alkane molecules were constructed with the Packmol program [43] and placed in a cubic simulation box of dimensions 50 × 50 × 50 Å^3^, corresponding to a system density of 0.1 g·cm^−3^. The all-atom GAFF force field [44] was employed, with atomic partial charges assigned using the AmberTools [45] suite to generate RESP2_0.5_ charge [46] files for each system. It is important to clarify that this value refers to the instantaneous local packing density during Packmol configuration generation, not the macroscopic thermodynamic density of the equilibrated simulation box. Following configuration generation, each system underwent a standard pre-equilibration protocol: energy minimization (steepest descent, 500,000 steps) followed by a short NPT [47] relaxation (P = 1 bar, T = 298 K, Berendsen barostat with τ_p_ = 0.1 ps) for 100 ps to adjust the box volume to physically consistent liquid densities (~0.72–0.75 g·cm^−3^). Only after full density convergence was achieved did we switch to the NVT ensemble for the 500 ps production runs described below. This two-stage protocol ensures that all reported production-phase densities in Table 4 correspond to properly equilibrated condensed phases at ambient pressure.

To validate the modeling approach, NVT [48] ensemble equilibration simulations were first conducted at 298 K with a time step of 1 fs for a total duration of 500 ps. Temperature control was achieved using the velocity-rescale (V-rescale) thermostat [49]. Convergence of the energy, temperature, and density profiles confirmed that the systems reached equilibrium. Subsequently, linear heating simulations were performed from 0 K to 2500 K at a constant heating rate of 50 K·ps^−1^ with a time step of 1 fs for a total duration of 1000 ps, during which temperature and density variations were recorded to preliminarily characterize the phase transition behavior of the four alkanes. All simulations employed periodic boundary conditions. Long-range electrostatic interactions were treated using the particle mesh Ewald (PME) method [50], and a cutoff radius of 20 Å was applied to van der Waals interactions.

### 3.3. xTB Molecular Dynamics

xTB molecular dynamics (xTB-MD) simulations [51] were carried out with the CP2K software package (version 2025.1) [52]. Simulation systems were prepared through a combined workflow using Packmol [43] and GROMACS: ten alkane molecules were placed in a cubic box of dimensions 30 × 30 × 30 Å^3^, maintaining a system density of 0.1 g·cm^−3^. Electronic structure calculations employed the semi-empirical GFN1-xTB method [18], which is based on the extended tight-binding approximation and offers a favorable compromise between quantum-chemical accuracy and computational efficiency, rendering it well-suited for dynamical simulations of pyrolysis processes in organic systems. Similar to the classical MD setup, this initial density represents the loose packing state during Packmol construction rather than the target thermodynamic density. For the xTB-MD simulations at 3500 K, the choice of a dilute starting configuration serves two critical physical purposes: (1) it accommodates the extreme thermal expansion and partial vaporization expected at this temperature, preventing unphysically high pressures during rapid heating that could cause integration instability or non-physical bond-breaking artifacts; and (2) it avoids steric overlaps and preparation-induced biases inherent in constructing dense initial configurations of flexible long-chain molecules, allowing the system to self-assemble into a physically realistic high-temperature state. The initial configurations were generated by randomly packing molecules using Packmol with independent randomization across all four systems, ensuring no systematic bias in molecular orientation or spatial distribution. The simulation box dimensions were held fixed during the subsequent NVT production run at 3500 K.

To address the critical question of method reliability, we have rigorously evaluated the applicability of GFN1-xTB for alkane pyrolysis simulations based on established benchmark literature rather than empirical assumption. Comprehensive assessments by Grimme et al. [18] and Bannwarth et al. [19] demonstrate that GFN1-xTB achieves mean absolute deviations (MADs) of ~4–6 kJ·mol^−1^ for reaction energies and <0.02 Å for equilibrium bond lengths of organic molecules relative to high-level DFT and coupled-cluster references. Crucially for this study, these benchmarks confirm that GFN1-xTB method reliably reproduces the relative energetics of C–C bond dissociation and radical stabilization across homologous series—precisely the physical quantities governing product selectivity in our xTB-MD simulations. While absolute activation barriers may carry uncertainties of ~10–15 kJ·mol^−1^ compared to canonical DFT, such systematic errors largely cancel when comparing trends within the C12–C15 series. Therefore, GFN1-xTB provides a physically sound compromise: it captures the essential electronic reorganization during bond breaking at a computational cost enabling nanosecond-scale sampling, while maintaining sufficient accuracy to resolve chain-length-dependent mechanistic differences. We explicitly frame all xTB-MD-derived kinetic conclusions as theoretically consistent trends within this validated accuracy envelope, rather than as absolute quantitative predictions. All electronic structure calculations employed both inner and outer SCF convergence thresholds of 1.0 × 10^–5^ Eh, with the Orbital Transformation (OT) method using FULL_SINGLE_INVERSE preconditioner, DIIS minimizer, and 2PNT line search algorithm. Ewald summation was enabled for long-range electrostatics (DO_EWALD T), and atomic charge checking was disabled (CHECK_ATOMIC_CHARGES F) to prevent spurious crashes during high-temperature bond-breaking events.

Furthermore, direct benchmarking of representative C–C cleavage channels (β-scission vs. terminal scission of sec-butyl radical) against B3LYP-D3/def2-TZVP confirms that GFN1-xTB correctly reproduces the relative energetic ordering despite a systematic absolute overestimation of ~58–71 kJ·mol^−1^, validating its reliability for qualitative mechanistic insights (see Table S2 and Figure S2 in Supplementary Materials). All xTB-MD simulations were performed in the NVT ensemble. Temperature was regulated using the canonical sampling through velocity rescaling (CSVR) thermostat [53] with a time constant of 100 fs. A time step of 0.2 fs was used, and a total of 30,000 steps were integrated, corresponding to a production run of 6000 fs (6 ps). Coordinates and velocities were saved every 10 MD steps (i.e., every 2 fs), providing sufficient temporal resolution for bond connectivity analysis. The simulation temperature was set to 3500 K, a value chosen based on the temperature at which significant alkane decomposition was observed in the classical MD heating simulations. Following energy minimization, the initial configurations were directly heated to the target temperature for dynamics propagation. Bond connectivity analysis was performed on a frame-by-frame basis using in-house scripts, with bond cleavage events identified based on interatomic distances: a cutoff radius of 1.8 Å was applied for C-C bonds and 1.2 Å for C-H bonds. Visualization and trajectory analysis were accomplished with VMD 1.9.3 [39].

## 4. Conclusions

This work establishes a multiscale theoretical framework integrating density functional theory, classical molecular dynamics, and semi-empirical molecular dynamics to investigate C12–C15 long-chain n-alkanes. The principal findings are as follows: (1) With increasing chain length, the narrowing HOMO-LUMO gap and decreasing ESP variance collectively reveal electronic “softening” and increasingly isotropic surface potential distribution. (2) Characteristic transition temperatures from classical MD simulations exhibit a monotonic increase with chain length that parallels experimental boiling point trends; despite a systematic absolute offset inherent to periodic boundary conditions, the consistent per-CH_2_ increment supports the reliability of the force field in capturing the cumulative scaling of dispersion interactions governing condensed-phase stability. (3) xTB-MD simulations definitively identify *β*-scission to ethylene as the dominant pyrolysis pathway, with longer chains yielding higher C2 fractions and greater retention of medium-sized fragments. We emphasize that these reactive simulation results represent early-stage, non-equilibrated observations rather than statistically converged pyrolysis product distributions; nanosecond-scale sampling would be required for quantitative yield predictions. Nevertheless, the integrated “electronic structure–condensed phase transition–initial reactive dynamics” perspective provided herein offers valuable theoretical insights and a methodological benchmark for future high-fidelity kinetic modeling of long-chain n-alkane pyrolysis.

## Figures and Tables

**Table 1:** Calculated results of chemical reactivity descriptors for the four kinds of long-chain *n*-alkanes.

Descriptors	n-Dodecane, Values (eV)	n-Tridecane, Values (eV)	n-Tetradecane, Values (eV)	n-Pentadecane, Values (eV)
E_LUMO_	0.79	0.79	0.79	0.78
E_HOMO_	−8.08	−8.05	−8.02	−7.99
Energy gap (∆*E*)	8.87	8.84	8.81	8.77
Ionization energy (*I*)	8.08	8.05	8.02	7.99
Electron affinity (*A*)	−0.79	−0.79	−0.79	−0.78
Electronegativity (*χ*)	3.65	3.63	3.62	3.61
Chemical potential (*µ*)	−3.65	−3.63	−3.62	−3.61
Global hardness (*η*)	4.44	4.42	4.41	4.39
Global softness (*σ*)	0.11	0.11	0.11	0.11
Electrophilicity (*ω*)	1.50	1.49	1.49	1.48

**Table 2:** Molecular Surface Electrostatic Potential and Energy Analysis for the four kinds of long-chain n-alkanes.

Molecular Name	n-Dodecane	n-Tridecane	n-Tetradecane	n-Pentadecane
Minimal value/kJ mol^−1^	−13.81	−13.99	−14.08	−14.19
Maximal value/kJ mol^−1^	28.54	28.54	28.53	28.48
Overall Average/kJ mol^−1^	8.41	8.39	8.38	8.37
Positive Average/kJ mol^−1^	13.68	13.66	13.64	13.60
Negative Average/kJ mol^−1^	−7.21	−7.30	−7.37	−7.45
Overall Variance/(kJ mol^−1^)^2^	57.66	57.08	56.59	56.18
Positive Variance/(kJ mol^−1^)^2^	42.93	42.11	41.35	40.87
Negative Variance/(kJ mol^−1^)^2^	14.73	14.97	15.24	15.31
Balance of charges (ν)	0.19	0.19	0.20	0.18
Internal Charge Separation/kJ mol^−1^	9.15	9.11	9.08	9.04
Molecular Polarity Index/kJ mol^−1^	12.05	12.06	12.07	12.07
Nonpolar surface area (|ESP| ≤ 41.84 kJ/mol)	100%	100%	100%	100%
Polar surface area (|ESP| > 41.84 kJ/mol)	0%	0%	0%	0%

**Table 3:** Statistical analysis of temperature parameters for C12–C15 long-chain n-alkanes during the production phase of molecular dynamics simulations.

Name	Average Temperature/K	Error Estimate/K	RMSD/K	Total Drift/K
C12	298.008	0.18	6.3092	−0.2533
C13	298.205	0.17	6.0206	−0.3537
C14	298.203	0.18	5.9872	−1.2834
C15	297.973	0.11	5.8138	−0.4252

**Table 4:** Statistical analysis of density parameters for C12–C15 long-chain n-alkanes during the production phase of molecular dynamics simulations.

Name	Average Density/(kg/m^3^)	Error Estimate/(kg/m^3^)	RMSD/(kg/m^3^)	Total Drift/(kg/m^3^)
C12	717.725	16	85.8474	97.7455
C13	730.485	14	76.2494	86.9867
C14	737.808	13	73.3007	70.2677
C15	748.503	12	62.2600	61.0273

**Table 5:** Statistical analysis of total energy parameters for C12–C15 long-chain n-alkanes during the production phase of molecular dynamics simulations.

Name	Total Energy/(kJ/mol)	Error Estimate/(kJ/mol)	RMSD/(kJ/mol)	Total Drift/(kJ/mol)
C12	8548.12	63	296.267	−397.646
C13	9363.22	73	287.504	−479.322
C14	9978.11	56	286.830	−235.006
C15	10,684.90	63	300.551	−314.536

## Data Availability

Data are contained within the article and Supplementary Materials.

## Supplementary Materials

The following supporting information can be downloaded at: https://www.mdpi.com/article/10.3390/molecules31132291/s1, Figure S1: Initial trajectory snapshots of the four compounds (C12–C15) taken at the beginning of the production phase after equilibration: (a) C12; (b) C13; (c) C14; (d) C15. All four panels show that the molecules are homogeneously distributed throughout the simulation boxes, confirming the robustness and rationality of the model construction for subsequent dynamic analyses; Figure S2: Comparison of relaxed potential energy profiles calculated at discrete bond-stretching intervals for representative C–C cleavage channels of the sec-butyl radical (sec-C_4_H_9_•) calculated at the GFN1-xTB and B3LYP-D3/def2-TZVP levels. (a) Energy profile along the β-scission pathway (sec-C_4_H_9_• → C_2_H_4_ + C_2_H_5_•) computed with GFN1-xTB; (b) energy profile along the terminal scission pathway (sec-C_4_H_9_• → CH_3_• + C_3_H_6_) computed with GFN1-xTB; (c) energy profile along the β-scission pathway computed with B3LYP-D3/def2-TZVP; (d) energy profile along the terminal scission pathway computed with B3LYP-D3/def2-TZVP. Both methods consistently predict lower reaction energies and barriers for β-scission compared to terminal scission, validating the qualitative reliability of GFN1-xTB in capturing C–C bond cleavage selectivity trends relevant to alkane pyrolysis; Table S1: Comparison of simulated characteristic transition temperatures (T_trans_) and experimental boiling points (T_b_, exp) for C12–C15 n-alkanes; Table S2: Benchmark comparison of reaction energies (ΔE_r_) for representative C–C cleavage channels of the sec-butyl radical calculated at GFN1-xTB and B3LYP-D3/def2-TZVP levels; S1: Statistical Analysis Methodology.
