# Supplementary material for: Study on Thermal Stability, Phase Transition Characteristics, and Pyrolysis Product Distributions of Long-Chain n-Alkanes (C12–C15)
Source: Molecules. 2026 Jul 1;31(13):2291. doi: 10.3390/molecules31132291 (PMC13362720; doi:10.3390/molecules31132291)
Supplement: Supplementary file 1 [file molecules-31-02291-s001.zip › molecules-4310028-supplementary.pdf]

molecules

MDPI

Supplementary Information

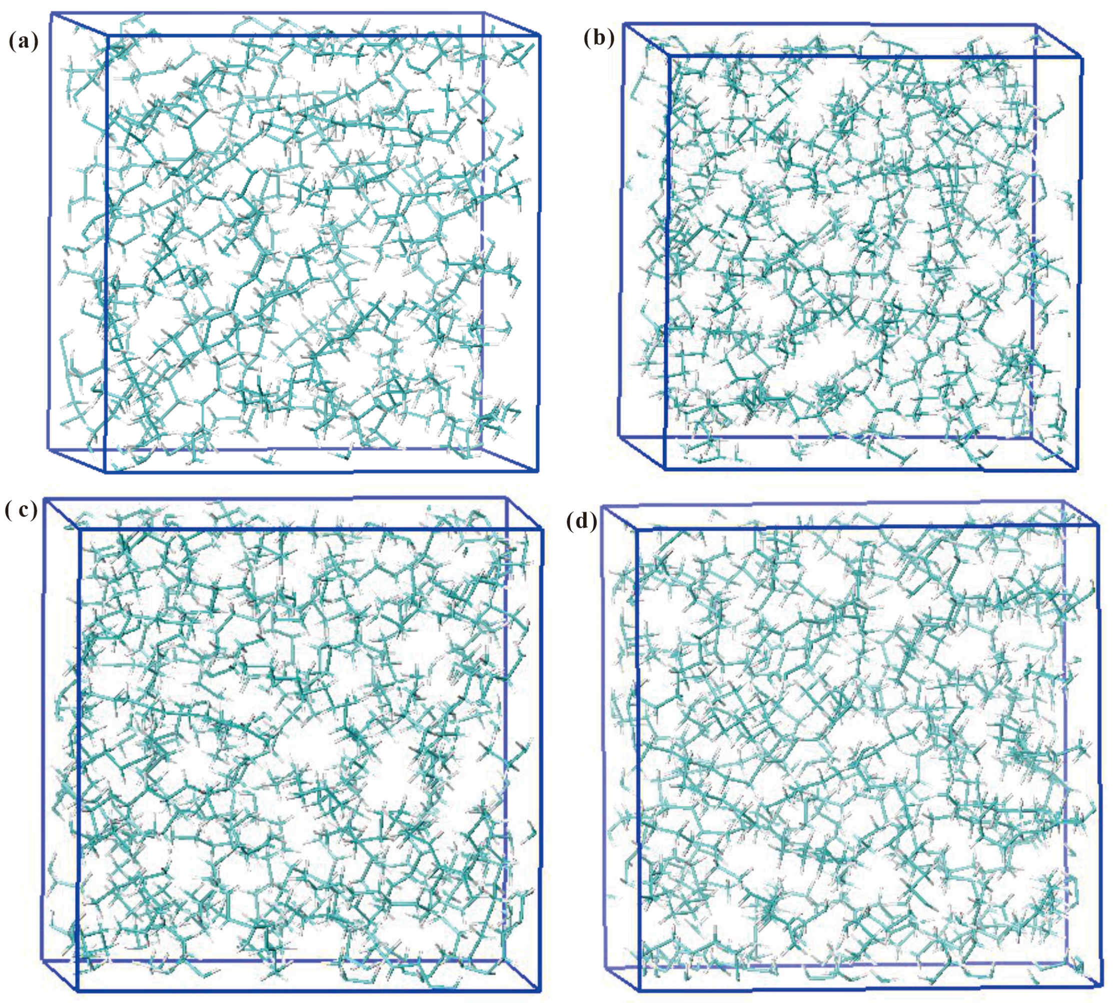

Figure S1. Initial trajectory snapshots of the four compounds (C12–C15) taken at the beginning of the production phase after equilibration: (a) C12; (b) C13; (c) C14; (d) C15. All four panels show that the molecules are homogeneously distributed throughout the simulation boxes, confirming the robustness and rationality of the model construction for subsequent dynamic analyses.

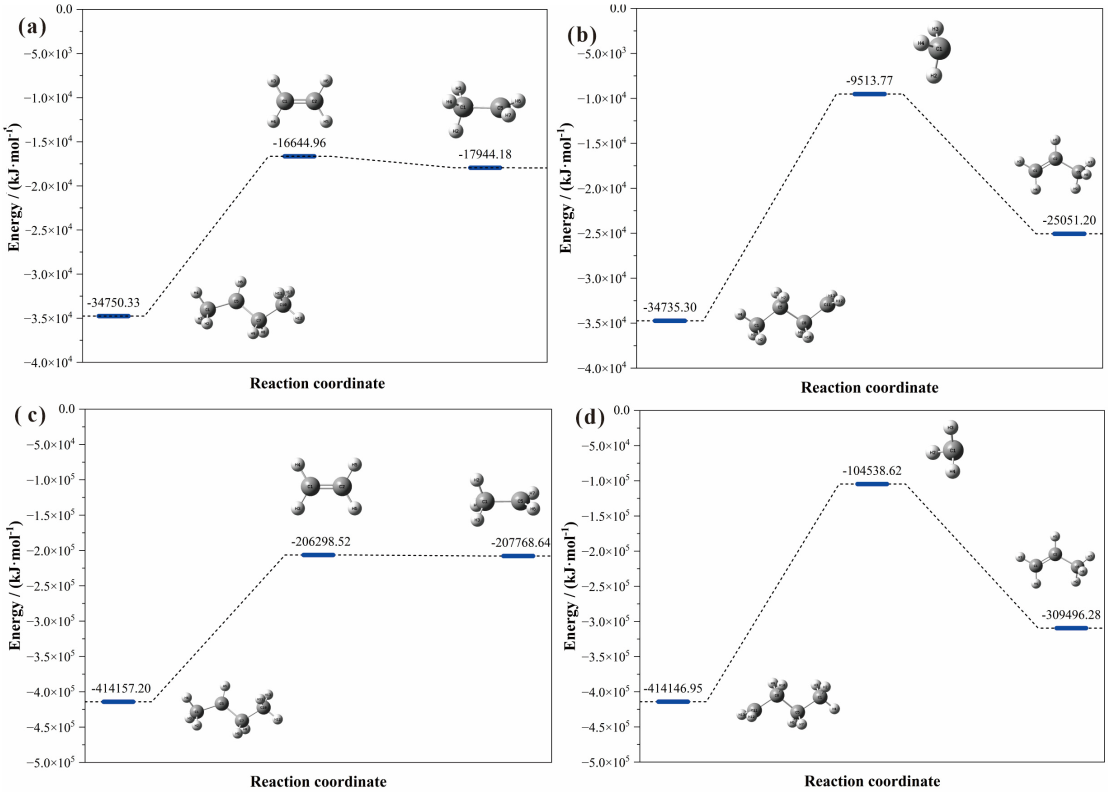

Figure S2. Comparison of relaxed potential energy profiles calculated at discrete bond-stretching intervals for representative C–C cleavage channels of the sec-butyl radical (sec-C_4H_9•) calculated at the GFN1-xTB and B3LYP-D3/def2-TZVP levels. (a) Energy profile along the β-scission pathway (sec-C_4H_9• → C_2H_4 + C_2H_5•) computed with GFN1-xTB; (b) energy profile along the terminal scission pathway (sec-C_4H_9• → CH_3• + C_3H_6) computed with GFN1-xTB; (c) energy profile along the β-scission pathway computed with B3LYP-D3/def2-TZVP; (d) energy profile along the terminal scission pathway computed with B3LYP-D3/def2-TZVP. Both methods consistently predict lower reaction energies and barriers for β-scission compared to terminal scission, validating the qualitative reliability of GFN1-xTB in capturing C–C bond cleavage selectivity trends relevant to alkane pyrolysis.

Table S1. Comparison of simulated characteristic transition temperatures (T_{trans}) and experimental boiling points (T_b, exp) for C12–C15 n-alkanes.

Names	C12	C13	C14	C15
Transition temperatures (T_{trans})/K	522	534	556	588
Experimental boiling points ($T_{b,\ exp}$)/K	489	508	526	543
Absolute deviations (ΔT)	–33	–26	–30	–45

Table S2. Benchmark comparison of reaction energies (ΔE_r) for representative C–C cleavage channels of the sec-butyl radical calculated at GFN1-xTB and B3LYP-D3/def2-TZVP levels.

Reaction Pathway	E_r(xTB) / kJ·mol^{-1}	E_r(DFT) / kJ·mol^{-1}	Deviation / kJ·mol^{-1}
β-scission: C_4H_9• → C_2H_4 + C_2H_5•	161.18	90.04	71.14
Terminal: C_4H_9• → CH_3• + C_3H_6	170.32	112.04	58.28
β vs Terminal Ordering	β < Terminal	β < Terminal	/

S1. Statistical Analysis Methodology

To quantify the reliability of the equilibrium properties reported in Tables 3–5, we employed standard statistical estimators over the production phase trajectories:

(1). Error Estimate (Standard Error of the Mean, SEM): The trajectory was divided into N non-overlapping blocks of equal length. The average value of each block was computed, and the SEM was determined as: $SEM = \frac{\sigma_{block}}{\sqrt{N}}$, where σ_{block} is the standard deviation of the block averages. This metric reflects the precision of the estimated mean.

(2). Root-Mean-Square Deviation (RMSD): The RMSD of a property X (e.g., temperature, density, or energy) measures its fluctuation amplitude around the mean $\overline{X}$:

RMSD = $\sqrt{\frac{1}{M}\sum_{i=1}^{M}(X_i - \overline{X})^2}$, where X_i is the instantaneous value at frame, and M is the total number of frames. This metric indicates the magnitude of thermal noise in the system.
